# Four Bad Habits of Modern Psychologists

**DOI:** 10.3390/bs7030053

**Published:** 2017-08-14

**Authors:** James Grice, Paul Barrett, Lisa Cota, Crystal Felix, Zachery Taylor, Samantha Garner, Eliwid Medellin, Adam Vest

**Affiliations:** 1 Department of Psychology, Oklahoma State University, Stillwater, OK 74078, USA; lisa.cota@okstate.edu (L.C.); crystal.felix@okstate.edu (C.F.); zachery.taylor@okstate.edu (Z.T.); samantha.garner@okstate.edu (S.G.); eliwid@okstate.edu (E.M.); adam.vest@okstate.edu (A.V.); 2 Department of Psychology, University of Auckland, Auckland 2120, New Zealand; paul@pbarrett.net

**Keywords:** inference, NHST, modeling, measurement, replication, Observation Oriented Modeling

## Abstract

Four data sets from studies included in the Reproducibility Project were re-analyzed to demonstrate a number of flawed research practices (i.e., “bad habits”) of modern psychology. Three of the four studies were successfully replicated, but re-analysis showed that in one study most of the participants responded in a manner inconsistent with the researchers’ theoretical model. In the second study, the replicated effect was shown to be an experimental confound, and in the third study the replicated statistical effect was shown to be entirely trivial. The fourth study was an unsuccessful replication, yet re-analysis of the data showed that questioning the common assumptions of modern psychological measurement can lead to novel techniques of data analysis and potentially interesting findings missed by traditional methods of analysis. Considered together, these new analyses show that while it is true replication is a key feature of science, causal inference, modeling, and measurement are equally important and perhaps more fundamental to obtaining truly scientific knowledge of the natural world. It would therefore be prudent for psychologists to confront the limitations and flaws in their current analytical methods and research practices.

## 1. Introduction

Written in the 13th Century, Questions V and VI of St. Thomas Aquinas’ *Expositio super Librum Boethii de Trinitate* can be considered as an early treatise on the philosophy of science [1]. In his commentary St. Thomas adopts and defends Aristotle’s view of science as essentially a unitary *habitus* of the human intellect that empowers a person to attain demonstrative knowledge of nature through its causes. To be clear, a *habitus* is not a tendency to automatically respond to some stimulus, as we might think today; rather, according to St. Thomas it is more like a disposition that empowers a person to accomplish some goal. Modern teachers of science understand quite well what St. Thomas was driving at in considering science to be a *habitus*. It is not enough, for example, to teach a student of geology the facts of the discipline. The student must also be taught *how to think geologically*. We can imagine St. Thomas hiking across the state of New Mexico. Via his theological *habitus* he might readily conclude that such beautiful and colorful terrain, so pleasing to the senses, can draw one closer to God. The student who has developed a *habitus* for geology, by comparison, might observe the same terrain, particularly the Sandia Mountains at sunset, and infer from their reddish color the presence of large quantities of Potassium-feldspar crystals. Such reasoning would evince the student’s capacity, or geological *habitus*, for actualizing a causal and scientific understanding of natural formations.

In the moral order bad habits are known as vices, and they can readily be observed in the reasoning, attitudes, and choices of persons who regularly violate societal norms of morality. In science, the identification of bad habits can be a more difficult task because the norms themselves may be problematic. In psychology, for instance, it has been repeatedly shown that Null Hypothesis Significance Testing is a fundamentally flawed framework for reasoning about and analyzing data; yet, this perfidious *habitus* remains entirely ubiquitous because it constitutes the norm by which journal editors, researchers, and educators judge the scientific merit of a given study or particular finding (see [2], for an erudite contemporary review that also advocates a “significance sameness” approach over NHST). As another example, replication is regarded as a hallmark of the scientific method, yet the normative practice among editors of psychological journals is to value novel research over exact replication studies, a fact brought to light by the recent Reproducibility Project [3] and controversial studies in psi phenomena [4,5,6].

In this paper, we present four additional, perfidious habits that may be preventing modern psychologists from developing truly scientific knowledge of human experience. Using studies from the aforementioned Reproducibility Project we expose and discuss each of these bad habits in turn. We also offer a potential remedy for each bad habit. As the materials and data sets from the Reproducibility Project are available in an open source library (https://osf.io/ezcuj/wiki/home/), they are ideal for these purposes. Moreover, the studies are recent, and therefore represent samples of the reasoning, methodology, and analysis techniques of modern psychologists. In other words, they provide an objective view of the modern psychological *habitus*.

### 1.1. Bad Habit #1: Inference Conflation

To make an inference is to use one’s power of reasoning to draw a conclusion. Two general forms of inference, induction and deduction, are widely known. A simple type of induction (reasoning from the specific to the general) found within the vast majority of published psychological studies is the inference from an observed sample statistic to an unobserved population parameter. This inference often occurs within the context of a Null Hypothesis Significance Test (NHST), in which the researcher judges a computed *p*-value to reach or exceed some low threshold (e.g., *p* ≤ 0.05). NHST itself is a highly problematic and unhealthy habit, but to make matters worse it can be shown that psychologists routinely confuse this inference for the inference they truly wish to make, which is the *inference to best explanation* (see [7,8]).

Schmidt and Besner [9], for example, investigated the causal mechanism underlying increases in the well-known Stroop effect. It has been known for some time that persons will typically take longer to identify the color of a word printed in an incongruous color (e.g., the word “blue” printed in the color red) than when the word is printed in the same color (e.g., “blue” printed in blue). The delayed reaction time is believed to be partly due to the effort required to separate the word from the color and ignore the former. Interestingly, when color-congruent words are shown with greater frequency across numerous trials of word-color pairs the Stroop effect increases. Schmidt and Besner argued that the best explanation for this phenomenon is a contingency threshold that is altered when frequencies of congruent words increase. The details of this mechanism need not be repeated here, but it is important to emphasize that the mechanism is meant to provide a causal account of the observed data. The mechanism is also understood to operate within the individual cognitive powers of each participant in the study, “... we test the hypothesis that *participants* prepare for a response by simply lowering the threshold for the expected response and do not alter the threshold for any other” (emphasis added, [9] p. 516).

This is a clear example of the type of inference most psychologists wish to draw from their research. First, a phenomenon has been observed; namely, higher frequencies of congruent word-color pairs in the Stroop task are associated with higher delays in identifying the printed color of subsequently presented words. Second, a mechanism has been proposed to explain why this phenomenon occurs, and the abductive argument can be expressed as follows:

Increases in the Stroop effect have been observed for different frequencies

 of congruent word-color pairs

If persons were to change thresholds when exposed to greater frequencies

 of word-color pairs, they would show increases in the Stroop effect

Therefore, persons changed thresholds

Third, the mechanism is put forth as offering a better explanation of the observed phenomenon than competing explanations; in this case, what Schmidt and Besner refer to as the modulation account [9] (p. 514). Lastly, as a general cognitive mechanism, it is understood to exist within the mind of any healthy, normally functioning adult, such as an undergraduate student (who was not randomly selected from some larger population to participate in a given study). The explanatory level is here the individual, not the interpersonal, societal, nor aggregate levels. In summary, the inference being sought is without question the inference to best explanation of an individual-level phenomenon.

**Figure 1:**
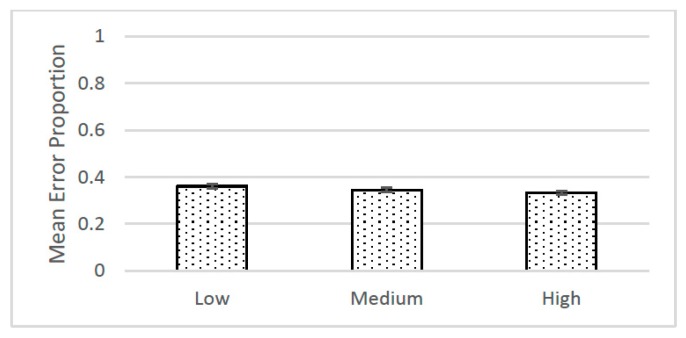
Means and standard errors for proportions of errors committed on the Stroop task for the low, medium, and high contingency conditions.

Following accepted practice (*habitus*!) with regard to research design and statistical analysis, however, Schmidt and Besner conflated this inference with the inference to population parameters. Specifically, each participant in the study was exposed to three levels of word-color pairings, referred to as low, medium, and high contingency conditions. The proportions of errors in the Stroop task were then tallied, averaged, and analyzed using a repeated measures analysis of variance and NHST. Based on whether or not the *p*-values were less than or equal to 0.05 the authors of both the original and replication [10] studies concluded that the three means were “significantly” different. The means and standard errors from the replication study are shown in Figure 1.

The inference from NHST for these studies was to population parameters, as is clear from the null hypothesis itself:

H_o_: μ_low_ = μ_medium_ = μ_high_

The μ’s represent population means for the three contingency conditions, and the hope is that the sample means represent unbiased estimates of these parameters—even though the population was never defined, nor were random samples drawn to help insure non-bias. The main point, however, is that the inference from the NHST had no necessary relation to the inference to best explanation the researchers were attempting to draw from their studies.

Eschewing NHST, the observed proportions of errors should have instead been examined at the level of the individuals. If the proposed mechanism is set into motion for each participant in the study, then each person’s proportion of errors should follow a simple ordinal pattern across the three contingency conditions; namely, low > medium > high. The proposed mechanism is not detailed enough to make explicit predictions about the exact magnitudes of the errors, but it clearly predicts such an ordinal pattern *for each person*. The question now becomes, how accurate is this ordinal pattern, which is based on the causal mechanism, when compared to the actual observations?

**Figure 2:**
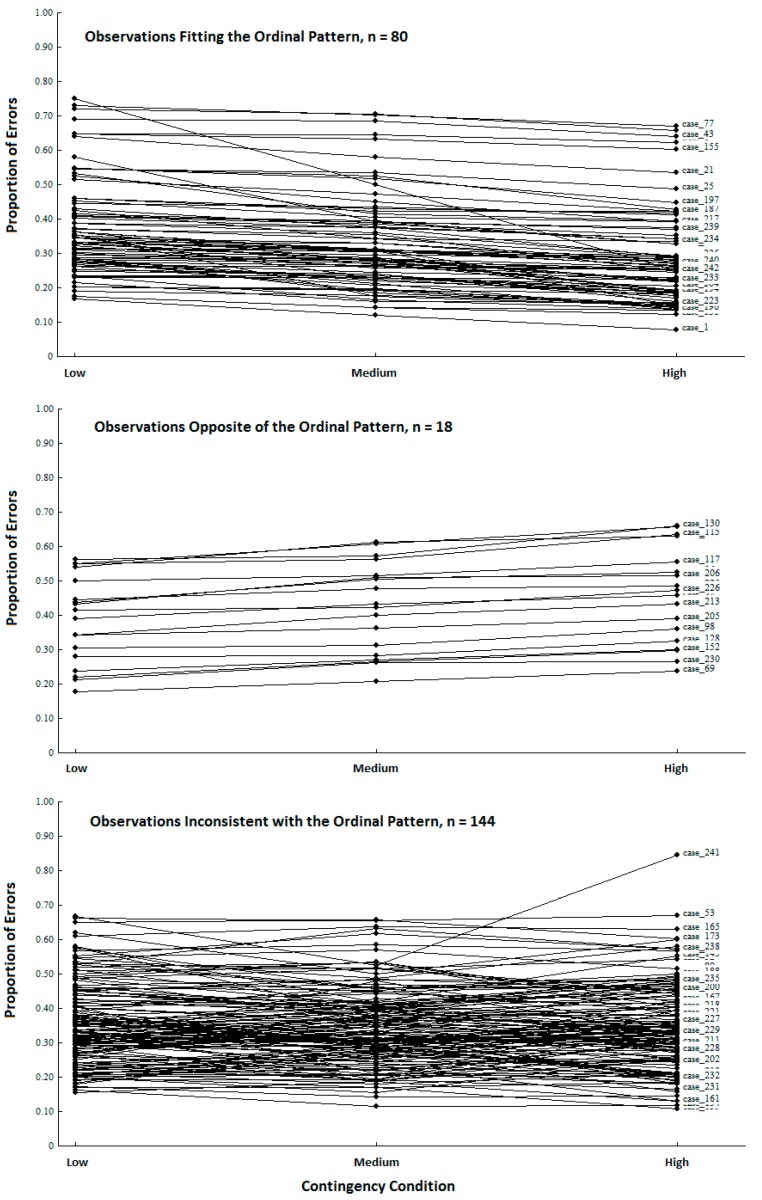
Proportions of errors committed on the Stroop task for the low, medium, and high contingency conditions. The results have been separated into three categories based on their ordinal patterns.

We used Observation Oriented Modeling [11,12] to analyze data from Cloud and Kyc’s replication study and discovered that the proportional errors for only 80 of the 242 persons (33.06%) fit the ordinal pattern. The proposed mechanism was therefore not operating as expected for a majority (66.94%) of the participants. Moreover, for 18 individuals (7% of the replication sample), the ordinal patterns of proportional errors were exactly *opposite* of the predicted pattern. The observations can be separated into the three general patterns shown in Figure 2: those who fit expectation (*n* = 80), those who showed the opposite pattern (*n* = 18), and those who were inconsistent with (but not opposite of) the expected pattern in some way (*n* = 144). With these results in hand, the inference to best explanation sought by the study authors is not generally supported by the data from the replication study. In other words, the proposed change in threshold across contingency conditions is not consistent with a majority of the persons’ responses. This conclusion was missed because NHST provides evidence for an inference which has no necessary bearing on a proposed causal mechanism operating at the level of the individuals in a given study.

### 1.2. Bad Habit #2: Anemic Modeling

**Figure 3:**
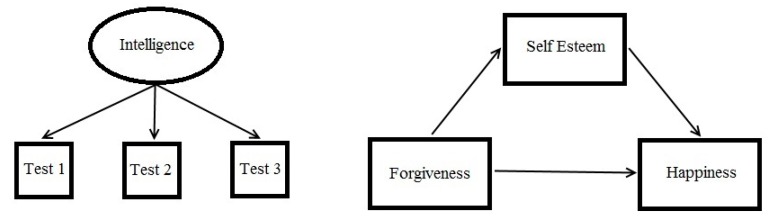
Example path diagrams showing a latent (ellipse) and observable (squares) variables.

There can be no greater ally to a scientist than a model. By constructing a model, the scientist produces a sort of picture of his or her reasoning about nature which can then be openly critiqued, modified, and systematically developed. The modeling *habitus* of most modern psychologists is embodied in path diagrams like those shown in Figure 3. As can be seen, such models provide simple visual summaries of theoretically proposed or empirically discovered relationships within sets of variables. Breiman [13] refers to these variable-based models as “data models” which are wedded to traditional experimental statistical methods (e.g., ANOVA, regression, factor analysis) and whose efficacy is therefore evaluated through *p*-values (NHST), effect sizes, goodness-of-fit indices, etc.

Compared to models found in the other sciences, particularly biological models such as the biochemical pathways of the eukaryotic cell, these data models are rather anemic. For instance, other than differentiating latent variables from observable variables, they provide no symbols for distinguishing between unique structures and processes comprising a natural system. Even a simple model of the atom exhibits different parts (neutrons, protons, electrons) and forces (spin, valence) organized into a coherent whole. Variable-based, data models also fail to provide the necessary framework for differentiating between the different types of causes (material, formal, efficient, and final causes; see [14]) that may be needed to explain human behavior. As Breiman [13] points out, these models are in fact not sufficient for representing the generative, causal mechanism underlying a set of observations. This task is left to “algorithmic models” which are not tied to traditional statistical methods and whose efficacy is evaluated on the basis of strict classificatory accuracy and theoretical coherence. Grice [11] refers to such models as “integrated models” in the domain of psychological research, and argues that insofar as the goal of science is to develop an accurate picture of the causal structure of natural systems, psychologists ignore these models at their peril. In more pragmatic terms, the modern data modeling *habitus* may be hindering the scientific progress of psychology.

The limitations of data modeling and the benefits of integrated modeling can be demonstrated by examining the study by Nairne, Pandeirada, and Thompson [15] which was replicated by Müller and Renkewitz [16] as part of the Reproducibility Project. In this study evidence for enhanced memory recall was discovered and attributed to “fitness-related” processing. With regard to specific procedures, volunteer undergraduate students were first asked to imagine themselves attempting to survive in the grasslands of a foreign land. With this image in mind, they were then asked to rate eight individual words with regard to their relevance to the imagined scenario. The rating scale ranged from 1 (irrelevant) to 5 (extremely relevant). Next, students were asked to imagine a scenario in which they were on an extended vacation at a fancy resort. Again, with this scenario in mind the students were asked to rate eight different words. The students were then asked to repeat imagining the two scenarios and to rate eight novel words for each scenario. For approximately one half of the students in Nairne et al.’s study the order of scenario presentation was reversed while the list of 32 words (arranged into four blocks) was held constant, yielding two groups: SVSV (survival-vacation-survival-vacation) and VSVS (vacation-survival-vacation-survival). Finally, after completing a filler task involving numerical digit memory and recall, the students were given a surprise recall task in which they were asked to recall from memory as many of the thirty-two previously rated words (16 in the survival condition, and 16 in the vacation condition) as possible.

Following the variable-based, data modeling *habitus* and drawing upon their understanding of fitness-related processing, Nairne et al. predicted that words rated in the survival scenario would be recalled with greater frequency (*p* ≤ 0.05) than words rated in the vacation scenario. In terms of a simple path model the two scenarios constitute the independent variable and the number of words recalled represent the dependent variable; hence, IV → DV (or, Scenario → Recall). Results from a repeated measures ANOVA supported the hypothesis, *F*(1, 23) = 5.70, *p* = 0.03, η^2^ = 0.20, with a higher proportion of survival-rated words (~53%) being recalled than vacation-rated words (~40%). These results were statistically replicated by Müller and Renkewitz: *F*(1, 31) = 12.13, *p* = 0.002, η^2^ = 0.28, survival proportion = 0.50, vacation proportion = 0.40.

**Figure 4:**
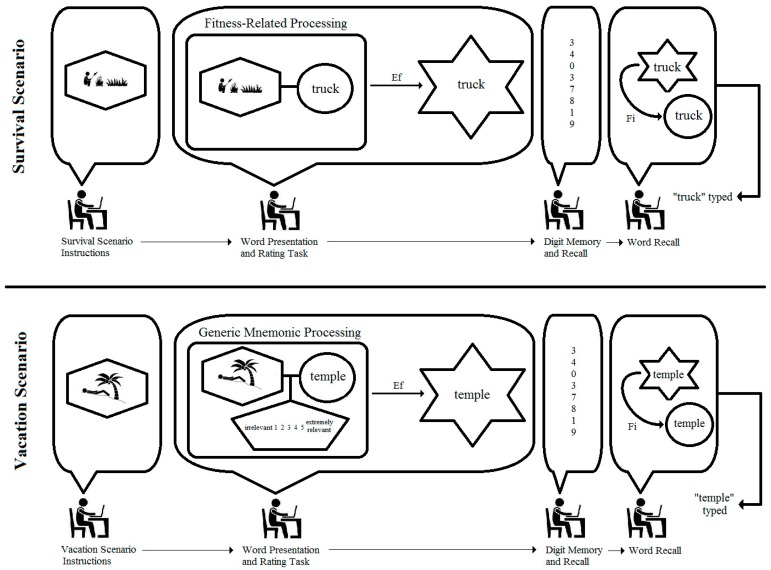
Model representing fitness-related and generic mnemonic processing of the words “truck” and “temple.” Visual images are represented as elongated hexagons; simple predication is represented as a circle; complex judgments are represented as pentagons; memory storage is represented as hexagrams; “Ef” represents efficient cause, and “Fi” represents final cause.

What is the generative mechanism within each student underlying the data and results from these two samples, and what alternative analyses might be conducted on the basis of a diagram of such a mechanism? The top panel of Figure 4 shows an integrated model for a male student who is asked to rate the word “truck” while imagining the survival scenario. As can be seen, the student is tracked through important phases of the experiment, and the structures and processes inferred in his conscious activity are represented as different geometric figures and arrows labeled with different types of causes. More specifically, the figure shows the student first reading the survival scenario instructions and imagining himself surviving on the grasslands. The imaged scene is presented in the elongated hexagon in the model. When presented with the word “truck” and asked if it is relevant to the scenario, the student is required to work simultaneously with two mental activities: (1) the imagined scenario, and (2) the predication of the word “truck” (represented as a circle in the model). Nairne et al. state clearly that the proximate mechanism of fitness-related processing is not known; hence, the model simply shows the two mental activities as interconnected and encapsulated by a rectangle labeled as “fitness-related processing”. The function of this processing is to enable the coding of the word “truck” into the memory store. This causal relation is shown as the arrow labeled “Ef” in the figure to denote it as an efficient cause, in distinction from a material, formal, or final cause (see [11,12,14]). The encoding process is represented by the hexagram. The student in the model is then shown completing the filler numerical recall task, and finally retrieving “truck” from the memory store and typing it as a response. The arrow labeled “Fi” indicates a final cause, as the student retrieves the word from memory for the purpose of fulfilling the experimenter’s request. The prediction from the model is clear: each student presented with “truck” in the survival scenario should recall the word at the end of the study. The same can be said for all the rated words in this scenario because the model is tantamount to a logical “if … then” statement. Simply put, if the word is subjected to survival processing, then it will be recalled.

It is of course well known that all scientific models are incomplete, and the integrated model in the top panel of Figure 4 is no exception. Other causal forces are surely operating within each student that might explain the recall of any given word, which is why the vacation scenario was included in the study. A model for this scenario for the same male student is presented in the bottom panel of Figure 4, and it shows “fitness-related processing” replaced with a “generic mnemonic process” component which entails the imagined vacation scenario, the predication of the word “temple”, and the judgment (elongated pentagon) of relevance on the 5-point rating scale. This latter judgement is not included in the top panel model of Figure 4 because it is regarded as inconsequential to fitness-related processing; and in a simple way, the second model provides a comparison of all other unspecified factors that serve as efficient causes of memory encoding. At the level of persons in the study, then, the specific prediction based on the models in the top and bottom panels of Figure 4 is that each student may or may not recall 100% of the survival-rated words, but regardless the observed percentage will be greater than the percentage recall of vacation-rated words.

Proceeding on the basis of these predictions, we re-analyzed Müller and Renkewitz’s replication data using the same ordinal analysis for the Stroop data above and found that only a small percentage of students recalled more words for the survival scenario, PCC = 52.63%. Using a randomization test [17], this was an unusual percentage, *p* = 0.03 (5000 random trials), but it’s magnitude does not invoke confidence in choosing the model in the top panel of Figure 4 over the model in the bottom panel. Importantly, the recall advantage for the survival scenario was only present in the VSVS condition, PCC = 63.16%, randomization *p* = 0.02 (5000 trials). The exact opposite pattern was found for the SVSV condition, PCC = 42.11, randomization *p* = 0.38. In other words, for the SVSV ordering, a majority of students recalled an equal or greater number of words for the vacation scenario compared to the survival scenario. These findings suggest a word/scenario confound that went unnoticed and uninvestigated by the replication team. Nairne et al. also failed to report analyses relevant to the potential impact of the two orders of stimulus presentation.

**Figure 5:**
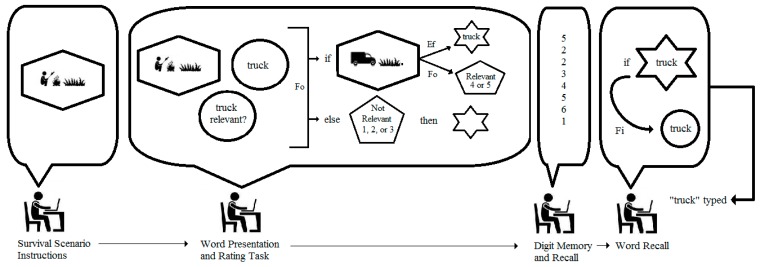
Model representing integration of “truck” into imagined scene of survival. Visual images are represented as elongated hexagons; simple predication is represented as a circle; complex judgments are represented as pentagons; memory storage is represented as hexagrams; “if” and “else” represent standard logical operators; “Ef” represents an efficient cause; “Fo” represents a formal cause, and “Fi” represents a final cause.

In order to replicate these controversial results and to explore the potential confound at the level of the persons (as Müller and Renkewitz presented only aggregate-level data), we recruited 99 undergraduate student volunteers to complete the exact same procedures used by the replication researchers. Again, the VSVS condition yielded an advantage for the survival-rated words (PCC = 77.55%, randomization *p* < 0.0002), while less than half of the students in the SVSV condition recalled more survival-rated words than vacation-rated words (PCC = 32.00%, randomization *p* = 0.92). The confound was thus replicated, and its specific nature can be unpacked by considering the competing integrated model in Figure 5. As can be seen, fitness-related and generic mnemonic processing have been replaced by a well-known memory mnemonic in which the word “truck” has been converted to an image and then integrated with the imagined survival scenario. Such integration is easily imaginable in this instance because a truck would obviously be relevant for surviving in tall grasslands as it would provide a means of transportation or escape. As noted by Nairne and Pandeirada [18] (p. 500), “If the relevance of the to-be-rated word to the survival scenario is immediately obvious … then there is little need to engage in much fitness-relevant processing”. The observable behavior of this relevance is the rating provided by the student. Consequently, the model in Figure 5 indicates another “if … then” relation: namely, if the word is judged as relevant because it has been integrated with the scenario, then it will be encoded in memory and recalled regardless of the content of the scenario. Again, the model is incomplete, providing no exact way to determine which scale values satisfy relevancy, but it nonetheless posits scale values (4 and 5) above the midpoint (3) as indicating relevance.

Which of the two explanatory models in Figure 4 (top panel) and Figure 5 offers the most accurate explanation of the recall frequencies? Simple tallies of each student’s responses based on logical statements can answer this question. If students rated words as 4 or 5 in either scenario, did they later recall those words? In answer to this question, there were 1188 (37.50%) words rated as relevant, and 565 of these words were successfully recalled (47.56%). By comparison, if words were presented in the survival scenario, did students later recall those words? In answer to this question, there were 1584 words (50% by study design) presented in the survival scenario, and 626 of these words were recalled successfully (39.52%). Analyzing these percentages on the individual level, a clear majority of the students (70.71%) recalled more relevant words than survival-rated words, PCC = 70.71, randomization *p* < 0.0002. The proportion was even greater for vacation-rated words, PCC = 81.82, randomization *p* < 0.002. These results indicate that the model in Figure 5 offers a more accurate explanation of the data than the fitness-related processing model in the top panel of Figure 4.

The nature of the confound above also becomes clear when working at the level of the persons in the study. Specifically, in the VSVS condition several words (hammer, shirt, sword, salmon, truck, horse) representing items or animals that could easily be integrated into a survival situation were matched with the survival scenario. In the SVSV condition, these words were matched with the vacation scenario in which they were not so easily integrated; e.g., what good would a hammer, sword, or truck be on a relaxing vacation at a fancy resort? Moreover, in the SVSV condition, two highly relevant words, “wine” and “ocean”, were matched with the vacation scenario. It is not surprising, then, that the expected pattern of recall was not supported in Müller and Renkewitz’s study or in the current replication effort for the SVSV condition. In brief, the findings supporting fitness-related processing appear to be due to a methodological confound involving a fortuitous pairing of words and scenarios.

The integrated modeling approach—or algorithmic approach using Breiman’s terminology—provided a more sophisticated set tools for conceptualizing Nairne et al.’s [15] study. By going beyond a verbal description or a simple path model connecting an independent and dependent variable in Figure 4 and Figure 5, we attempted to make explicit the different structures and processes underlying the responses of individuals completing the experimental tasks. Within these figures we drew distinctions between different mental events and processes (e.g., predication, imagination, memorizing) as well as different causes (e.g., formal and final causes), thus offering a picture of reality that is richer and more theoretically satisfying than one could ever hope to achieve with a set of interconnected ellipses and boxes in a path diagram. As shown above, integrated models also provide the means for comparing specific, competing causal claims, therefore creating a propitious environment for constructive debates regarding theory, research design, measurement, etc. Moreover, as causal mechanisms routinely operate at the level of the individuals in studies of psychology, data analysis is similarly person-centered rather than aggregate-centered, as demonstrated above. Old habits of judging results based on NHST, residual analysis, and effect sizes thus fade into to the background while a new *habitus* of judging results primarily on the basis of accuracy takes hold.

### 1.3. Bad Habit #3: Much Ado about Nothing

One of the advantages of the model comparisons approach toward analysis of linear modeling [19] is that it incorporates the popular notion of parsimony. Strictly speaking, the principle of parsimony states that “entities are not to be multiplied beyond necessity”, but as commonly understood among scientists, it entails a preference for modeling natural phenomena in the simplest possible terms. In multiple regression, for instance, a model with fewer predictors would be preferred over a model with numerous predictors if the latter yielded a negligible or trivial increase in explanatory power. Unfortunately, the modern *habitus* for determining whether a more complex model is indeed preferable involves NHST (i.e., indexed by the *p*-value) rather than a thorough assessment of the model’s increased explanatory power or increased predictive accuracy. Using simulations based on genuine studies, Barrett [20] recently demonstrated the detrimental outcomes of this bad habit, showing that psychologists are apt to report incremental effects as both scientifically informative and pragmatically important when in truth they are trivial and meaningless.

The validity of Barrett’s arguments can be verified by examining the study by Payne, Burkley, and Stokes [21] which was replicated by Vianello [22] as part of the Reproducibility Project. In this study, the relationship between direct and indirect assessments of racial bias were examined. Specifically, undergraduate students were presented with counterbalanced images of Black and White male faces on a computer screen. In the *indirect* phase of the experiment each face was followed by the presentation of a Chinese pictograph which the students were asked to rate on a 4-point scale: (−2) very unpleasant, (−1) slightly unpleasant, (+1) slightly pleasant, and (+2) very pleasant. The students’ responses to the scale were averaged separately for the Black and White faces, and then differences were computed between these averages. For any given student, a positive difference was interpreted as indicating an average *implicit* (i.e., *indirect*) preference for White faces, while a negative difference indicated an average implicit preference for Black faces. A total of 12 Black and 12 White faces were used, but each face was paired twice with a unique pictograph. In the *direct* condition of the experiment, the students were asked to rate the faces themselves rather than the pictographs. For this condition, however, the 12 Black and 12 White faces were only presented once. Each student was exposed to the *indirect* and *direct* conditions (counterbalanced across students), and the original study authors expected a moderate correlation between the two sets of ratings. In terms of a regression equation, the indirect ratings were expected to be moderately predictive of the direct ratings: y*_direct_* = *a* + *b*x*_indirect_* + ε.

Payne et al. went on to hypothesize that the magnitude of this predictive relationship could be manipulated by applying social pressure. Consequently, approximately one half of the students were instructed to specifically avoid racial prejudice prior to making their direct and indirect ratings. The other students were instructed to express their own opinions and attitudes as honestly as possible prior to making their ratings. These two groups of students were referred to as *high-* and *low-pressure*, respectively; and as a result of the manipulation, “we predicted a strong implicit-explicit correlation only in the low-pressure condition, where participants could feel free to overtly express their attitudes” [21] (p. 27). In other words, an interaction was expected and tested by first adding the grouping variable to the original equation,

y*_direct_* = *a* + *b*_1_x*_indirect_* + *b*_2_x*_group_* + ε, (1)

and then adding an interaction term,

y*_direct_* = *a* + *b*_1_x*_indirect_* + *b*_2_x*_group_* + *b*_3_x_indirect*group_ + ε. (2)

The key statistical prediction using the model comparisons approach was that, above and beyond the indirect and group main effects, the 2-way interaction would result in a significant (i.e., *p* ≤ 0.05) increase in the variance explained for the direct ratings. Payne et al. [21] indeed reported a statistically significant increment in R^2^ (ΔR^2^ = 0.09, *p* < 0.05), and Vianello replicated this significant finding, showing that the interaction term increased multiple R^2^ from 0.297 to 0.313 compared to the model with only main effects (ΔR^2^ = 0.016, *p* < 0.05).

Using NHST as the sole arbiter of whether a finding has been replicated (or not), ignores the fact that the magnitude of effect being addressed may not be theoretically or practically meaningful. In general terms, the purpose of Payne et al.’s [21] study was to investigate how individuals’ explicit and implicit biases towards minorities might be related. In other words, are the two biases connected within the psyche of each person so that one might be predicted from the other? Moreover, can this connection be modified—and the prediction of explicit bias made more accurate—via experimental manipulation? These questions can be combined and restated in terms of the regression models above; specifically, does the interaction model (Equation (2)) show a meaningful improvement in accuracy for predicting individuals’ y*_direct_* values compared to the more parsimonious main effects model (Equation (1))? This question places emphasis on the individuals in the study as well as the original units of observation for bias (viz., the −2 to +2 rating scale), which is appropriate because explicit or implicit racial bias is a deeply personal experience, and the proxy to that experience in this study is the rating scale. Analyses should consequently eschew aggregate ΔR^2^ and *p*-values from NHST and instead provide an individual-level assessment of the predictive accuracy of the regression models in the original units of observation. Only in this way can the theoretical or practical meaningfulness of the results be assessed adequately.

**Figure 6:**
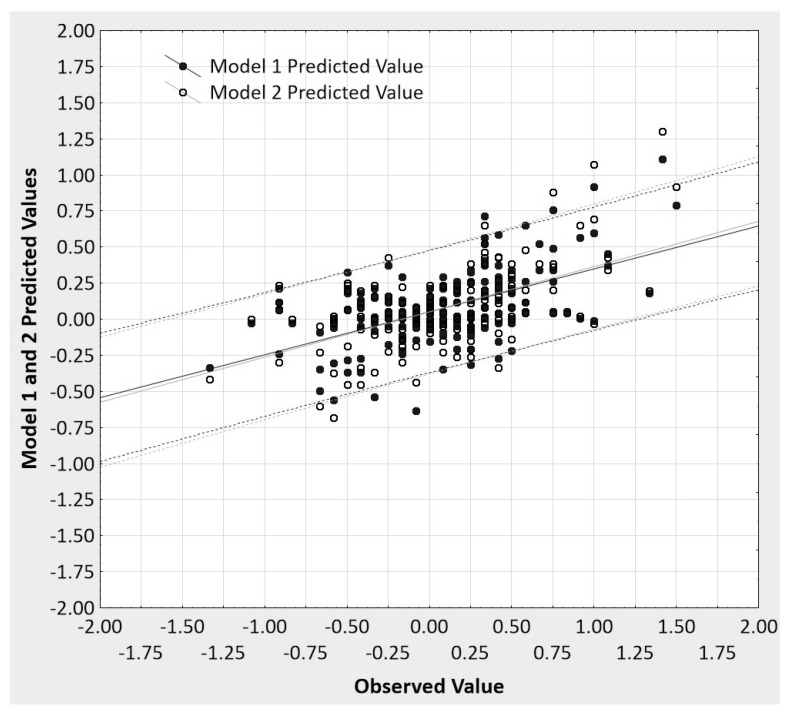
The observed vs. predicted values for each regression model, the two regression lines, and the 95% prediction intervals around each regression line (the dotted lines).

Toward that end, Figure 6 presents the observed values plotted against the predicted values for each model, the two regression lines, and the 95% prediction intervals around each regression line for the data from Vianello’s [22] replication study. A serious attempt at establishing *predictive accuracy* of any regression uses prediction, not confidence intervals, or it bootstraps the appropriate prediction intervals from V-fold or some other form of cross-validation of regression results. The prediction interval gives information on individual predictions of the dependent variable, while a confidence interval is about estimating a likely mean (aggregate) prediction. That is, a prediction interval for a predicted value of the dependent variable gives us a range of values around which an additional observation of the dependent variable can be expected to be located with a given level of certainty. In contrast, the confidence interval provides the estimated range of values within which a certain percentage of all confidence intervals computed from independent samples from the same population will include the unknown population parameter (the mean predicted value). As can be seen in Figure 6, the two models’ predicted values are barely discriminable from one another, likewise the regression lines and prediction intervals. Moreover, the prediction intervals are so wide as to suggest that making any strong claims based upon these data is unwise.

**Figure 7:**
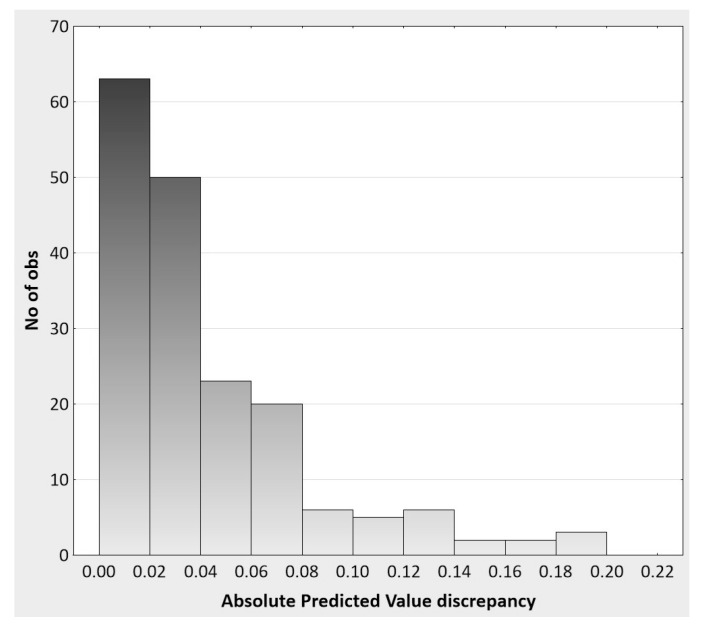
The histogram of absolute value discrepancies between Model 1 and Model 2 predicted observations.

The trivial difference between the two regression models can also be shown by investigating the degree of predictive ‘lift’ of the interaction model over the main effects model. Again, this analysis is based on the actual metric (and rating scale range of −2 to +2) of the observations at the level of the individuals in the replication study. Specifically, the absolute discrepancies between the two regression models in terms of their predictive accuracy of y*_direct_* are summarized as follows: *n* = 180, *mdn* = 0.023, *min* = 0.002, *max* = 0.200, 10th *percentile* = 0.005, 90th *percentile* = 0.102. Given minimum and maximum possible discrepancies of 0 and 4 on the rating scale, respectively, the median discrepancy of 0.023 shows a 0.58% lift in predictive accuracy when the interaction term is included in the regression model. The histogram in Figure 7 moreover shows that the absolute discrepancies for most of the participants (63%) are 0.04 or less, and 71% of discrepancies are less than 0.05, thus demonstrating the inconsequential impact of adding the interaction term to the main effect regression model. It is difficult to imagine how these results are theoretically or practically meaningful when considered at the level of the person. Picture, for instance, a male student named Nathan with a y*_direct_* score of 1.5 (range: −2 to +2) and a predicted score of 1.2 from the main effects regression model. Suppose further that by adding the interaction term, Nathan’s predicted score increases by 0.023 (the median discrepancy) to a value of 1.223. How can this difference reflect anything remotely meaningful in Nathan’s subjective experience as it pertains to the modifiability of the connection between his implicit and explicit racial biases? This is the central question that is left entirely unanswered by the modern model comparisons *habitus* which focuses on aggregate ΔR^2^ values and NHST, and which leads to the unwarranted acceptance of overly complex (i.e., less parsimonious) models and trivial statistical effects in psychological research.

### 1.4. Bad Habit #4: Measurement and Washing Brains

Just as John Quincy Adams once opined that weights and measures are “necessary to every occupation of human industry” [23], one could argue today that the act of measurement is central to the scientific method. What exactly does it mean, however, to measure something in nature? For psychologists, the modern *habitus* is to regard measurement as (a) the assignment of symbols or numbers according to specified rules, and (b) the classification of phenomena according to the four scales of measurement: nominal, ordinal, interval, and ratio. The human quality *gender*, for instance, is regarded as measurable using a nominal scale (e.g., assigning ‘M’ or ‘F’ for male and female, respectively), whereas the quality *intelligence* is considered as measurable using an interval scale (e.g., recording individuals’ total scores from the WAIS).

Unfortunately, what has been lost to history is the fact that the definition of measurement above, as well as the four scales of measurement themselves, were invented by S.S. Stevens in 1946 in response to claims that psychologists had not successfully established units of measure for the psychophysical attributes (e.g., perception of brightness or sound) they were studying in the early 1900s. These claims came from members of a committee appointed by the *British Association for the Advancement of Science* in 1932 to investigate the measurement practices of psychologists. According to Michell [24] (p. 15), a number of committee members understood measurement—at least implicitly—as “the discovery or estimation of the ratio of some magnitude of a quantitative attribute to a unit (a unit being, in principle, and magnitude of the same quantitative attribute)”. In other words, to measure successfully a scientist must establish arbitrary units that correctly represented the continuous quantitative structure (i.e., the additive structure) of the attribute under investigation. Acknowledging the committee could not unanimously agree that psychologists had produced additive units of measure, Stevens sought to clarify what he regarded as a conceptual rather than scientific problem by offering his own definition of measurement as “the assignment of numerals to object or events according to rules.” [25] (p. 677). His definition was decidedly subjective as it reduced measurement to a simple question, “What are the rules, if any, under which numerals are assigned?” [25] (p. 680). Two personality psychologists, for instance, using two entirely different rating scales (e.g., dichotomous versus multi-point rating scales) and unique weighting schemes for their respective self-report questionnaires could both be said to have successfully measured *introversion* per Stevens’ viewpoint. Whether or not *introversion* is a human attribute that is structured continuously and therefore measurable in the sense defined by Michell above is a question that is simply never addressed nor raised. Indeed, nearly 100 years after Carl Jung [26] first introduced *introversion* as a personality trait, no unit of measure has been developed despite countless self-report questionnaires utilized daily by psychologists around the world.

The long-term impact of Stevens’ subjective formulation has been the topic of spirited debate (e.g., see [27,28,29]), but there can be no doubt the vast majority of modern psychologists have adopted his views; and because of their desire for the statistical power that comes with parametric data models and analyses, they often presume to be working with interval or ratio scaled measurements. In other words, without empirical, scientific justification they assume they are working with established units of measure for continuously structured quantitative attributes. An example of how this *habitus* operates in the realm of psychological research can be seen in Exline et al.’s [30] study on self-reflection and vengeance which was replicated by Lin [31] as part of the Reproducibility Project. The overall goal of the original study was to demonstrate that men would be less likely to seek revenge on another person if they were first asked to reflect upon their own capacity for wrongdoing. Previous research had demonstrated a statistical effect for men, but not for women, in this regard.

For the replication study, 135 individuals (40.74% male) completed several self-report questionnaires. The participants first wrote about an experience in which they were offended, harmed, or hurt by another person. In one condition, they then responded to a series of questions regarding their personal capability to commit an act of wrongdoing; e.g., “Can you imagine a situation in which you could do something as bad as what the other person did?” For the primary dependent variable, the participants then completed the 18-item *Transgression Related Interpersonal Motivations* (TRIM) inventory (example items are “I’ll make him/her pay” and “I am avoiding him/her”). Responses were made on a 5-point Likert-type scale (1 = *Strongly Disagree*, 2 = *Disagree*, 3 = *Neutral*, 4 = *Agree*, 5 = *Strongly Agree*) which were averaged to form a total TRIM score and three subscale scores: Avoidance Motivations, Revenge Motivations, and Benevolence Motivations. In a second condition, participants completed the same self-report questionnaires, but the TRIM inventory was completed *before* the items regarding personal capacity for wrongdoing. Based on results from the original study, the replication author expected men to show lower scores for the Revenge Motivations subscale of the TRIM when they completed the personal capacity items first compared to when they completed those items second. In other words, men who first reflected on their own capacity for wrongdoing were expected to be more forgiving, on average and in some unspecified population (using NHST with *p* ≤ 0.05), than men who did not self-reflect. The results from this particular replication study were not statistically significant (*p* > 0.05) and thus failed to confirm the original findings.

Was it legitimate, however, to average responses from the TRIM inventory? The justification for computing the total and subscale averages rests entirely upon the modern measurement *habitus*. Specifically, the rating values are presumed to be interval scaled data, using Stevens’ four scales of measurement. As is well known a wide variety of mathematical and statistical operations can be performed on interval data, such as addition, subtraction, and averaging [32]. With ordinal or nominal data, the number of operations is greatly restricted. Consider a participant named Jonathan who responded to the “*I’ll make him/her pay*” item as follows:
Strongly
StronglyDisagreeDisagreeNeutralAgreeAgree123④5

Unfortunately, there is no scientific evidence that the values 1 through 5 represent units of measure for the attribute *revenge motivation*, which is presumed to underlie the participants’ responses. Following Dorough-Morris et al. [33], this can be made plain by examining a similar item marked by Jonathan designed to “measure” an attribute with well-known units of measure:
*I have a lot of height*Strongly
StronglyDisagreeDisagreeNeutralAgreeAgree123④5

In what way does the chosen value of 4 on the scale reflect Jonathan’s physical height? Is Jonathan 6’10” which would be considered relatively tall among men, or is he 5’10” which he would personally consider as tall because most of his friends are 5’5”? Furthermore, suppose Mark rates himself as 3 and Robert rates himself as 2 on the tallness item. Taking differences, can we conclude the difference in *tallness* (i.e., height) between Jonathan and Robert (4 − 2 = 2) is twice the difference in *tallness* between Jonathan and Mark (4 − 3 = 1)? Such operations are only legitimate if we have established units of measure for the *tallness* attribute, which as pointed out by Michell [24] entails demonstrating that *tallness* itself possesses additive properties. Clearly, the US Customary and Metric systems provide units of measure for height whereas the 5-point Likert-type scale does not. Computing differences, adding values, and taking averages both within and between Likert-type rating scales are therefore questionable practices.

Classic psychometric theory presents an even deeper problem regarding the TRIM scores. Suppose Jonathan responds as follows to another Revenge Motivations subscale item, “*I wish that something bad would happen to him/her*”:
Strongly
StronglyDisagreeDisagreeNeutralAgreeAgree1234⑤

The vengeance subscale is scored by averaging Jonathan’s responses to the two items shown herein and three others on the questionnaire. The ostensible payoff promised by the Classical True Score Model [34] of psychometric theory for computing an average is greater reliability and validity. According to the CTSM, each of Jonathan’s responses is an error-laden measure of his true level of perceived *revenge motivation*. Moreover, the errors in the measures are assumed to be independent and random; hence, by computing an average of the responses, the errors are given the opportunity to cancel each other out, yielding a mean score that is relatively less saturated with error than each of the individual items. However, even if units of measure are granted for the Likert-type scale, there is simply no good reason to assume that Jonathan’s responses—and therefore his errors—are independent. Lord and Novick [34] acknowledged this problem approximately 50 years ago in arguing that test constructors would have to assume their respondents were undergoing some sort of brainwashing between items in order to delete their memories. For Jonathan, for example, we would “wash his brains” (quoting [35] (p. 29)) after each response to an item on the TRIM. After a small amount of deliberation, Lord and Novick [34] judged the usefulness of the CTSM to be worth the risk of making such a strange and unwarranted assumption. With no units of measure and dependence among observations, however, the critical-minded scientist is left wondering about the exact meaning of the Revenge Motivations subscale scores in Exline et al.’s [29] original study and Lin’s [31] replication.

Fortunately, the modern measurement *habitus* need not be adopted to examine the Likert-type rating scales incorporated in the TRIM. The first step away from old habits is to conceptualize the responses as ordered categories in which the scale represents a series of judgments that can move from a state of certain disagreement to a state of certain agreement with the provided statement. This process is demarcated into five categories from which the participant must choose as representing his or her own thoughts. The responses are therefore not independent mini-measurements using established units of measure, but rather constrained snapshots of the person’s subjective experience when reflecting upon an instance of wrongdoing. Consequently, the TRIM data from this study should not be analyzed in a way that presumes to cancel out error via the computation of averages, but instead examines patterns within the participants’ responses taken “as is”.

Using a *Threshold Analysis* [36], which is a nonparametric alternative to logistic regression, we sought to derive a logical rule for discriminating between the men who completed the personal capability items prior to or after completing the 18 items on the TRIM. This analysis works by first identifying a threshold value on the 5-point Likert-type scale of each item that provides maximal discrimination between men in the two groups (i.e., between those who completed the TRIM first and those who completed it second, after the personal capability items). For example, 67.27% of the men could be correctly classified into their respective groups when the item “*Despite what he/she did, I want us to have a positive relationship*” was divided into two ordered categories: *Strongly Disagree* and *Disagree* versus *Neutral*, *Agree*, and *Strongly Agree*. All 18 items were dichotomized per their respective thresholds. The analysis then formed logical combinations of these dichotomous orderings (variables) to maximally discriminate between the two groups of men, and the following combination produced the greatest discriminatory value:

[Item 4_(3:5)_ ˅ Item 16_(3:5)_] ˄ Item 18_(1:4)_

The items are as follows:

- Item 4: *I wish that something bad would happened to him/her*
- Item 16: *I have released my anger so I can work on restoring our relationship to health*
- Item 18: *I withdraw from him/her*

The subscripted values in parentheses indicate the range of scale values endorsed on the items (1 = *Strongly Disagree*, 2 = *Disagree*, etc.), and the ∨ and ∧ symbols represent logical disjunction and conjunction, respectively. Men who first reflect upon their own capacity for wrongdoing were more likely to respond in ways consistent with this logical expression than men who did not self-reflect. The logical statement suggests men in the former group were not completely withdrawing from the perpetrator of the harmful act (Item 18 endorsed *Strongly Disagree* to *Agree*), but in so doing they found themselves faced with the choice of wishing ill upon the person (Item 4 endorsed *Neutral* to *Strongly Agree*) or giving up their anger toward the person (Item 16 endorsed *Neutral* to *Strongly Agree*). Remarkably, the patterns of responses for 28 of the 31 men (90%) who first reflected on their own capacity for wrongdoing matched this logical statement, whereas the responses for 12 of the 24 men (50%) who did not self-reflect matched. The overall Percent Correct Classification (PCC) was 72.73%, and a randomization test based on the data from all 55 men showed this result to be highly improbable (*p*-value = 0.001 for 1000 randomized trials). By comparison, the PCC index for women in the sample was only 56.25%, randomization *p*-value = 0.38.

Contrary to the negative results reported in the replication study, then, vengeful thoughts may be alterable by asking men to self-reflect on their own capacity for wrongdoing—but only if these thoughts are considered contextually or holistically. In other words, rather than imagining men to have their brains continually washed between item responses in an ill-conceived bid to cancel out measurement error, it may be more fruitful to imagine men reasoning their way through a set of tasks in which they are asked to integrate various ideas relevant to avoidance, revenge, and benevolence. The discriminating logical statement discovered above, which of course should be scrutinized and replicated in an independent sample to be taken seriously, was only made possible by adopting the latter viewpoint; and this viewpoint itself was only made possible by recounting the history of psychological measurement and revealing the source of the modern measurement *habitus*.

## 2. Discussion

Repeatability of both method and findings is a hallmark of scientific knowledge. The recent efforts to expose, discuss, and correct the replication crisis in modern psychology are therefore to be applauded. However, as pointed out by Ferguson [37], the historic disregard for replication in psychology is not the only tarnish on its reputation as a science. A variety of methodological, theoretical, and cultural problems also plague the discipline, many of which were identified nearly 30 years ago in Lykken’s [38] book chapter, *What’s Wrong with Psychology Anyway?* Woods’ [39] revisited Lykken’s laundry list of problems and discovered that, consistent with Ferguson’s assessment, very little has been done to improve the science of psychology.

Our examination of the four studies above follow in this tradition of identifying important problems with psychologists’ research practices. By selecting studies from the Reproducibility Project [3], our analyses moreover demonstrate these problems are not abstract and inconsequential, but rather impact the very conclusions drawn from contemporary psychological research. It could be argued that the bad habits identified in this paper are in fact more fundamental than replication. Three of the four studies selected were successful replications of prior research. But what, exactly, was replicated?

In the first study, our analyses showed that most of the persons in the sample did not respond to the Stroop task in a manner that was consistent with the theoretical model. Moreover, 7% of the individuals in the study responded in a manner exactly opposite of expectation. These important findings were missed because of the unwarranted centrality of Null Hypothesis Significance Testing (NHST) and the confusion it brings to research practice. In the second study regarding enhanced memory, a difference between experimental groups was missed in the replication analysis, but identified in our own replication sample of participants. More importantly, a competing causal model based on a well-known memory mnemonic was created, tested, and found to offer a more accurate explanation of the patterns within the replication data. These findings were overlooked because modern approaches to diagramming models and reasoning about psychological phenomena are too homogeneous and simplistic to accurately reflect psychological phenomena. Lastly, in the third study the “effect” successfully replicated was shown to be entirely trivial when the data were analyzed in such a way as to focus on the units of observation employed by the researchers. The trivial effects were originally made to appear important through NHST-based analyses and the somewhat lax interpretation guidelines employed by psychologists worldwide.

We chose the fourth study regarding self-reflection and vengeance partly because it was a failed replication, which permitted us to ask “what findings, potentially, are being missed by psychologists using contemporary methods?” After the tradition of summing or averaging responses to self-report inventories was questioned, a different way to view the data was adopted. Novel analyses consequently revealed a potentially interesting pattern of observations which suggested men could be impacted by a self-reflection task prior to considering how they may or may not seek revenge on a person who had committed a wrongful act against them. The pattern was logical in nature, presenting a picture of the men as reasoning agents attempting to make sense of the imagined situation of wrongdoing and to develop an appropriate plan of action. The novel approach embodied in the new analyses therefore provided a less mechanistic view of the men in the study.

Consistent with Ferguson’s [37] stated purpose for presenting his list of problems plaguing modern psychology, the bad habits above are presented in the spirit of optimism. For each habit, a set of reasoning and analysis tools was also presented to help psychologists improve their research. Regarding the first habit, it is now widely acknowledged that NHST should never have ascended to the position of gatekeeper for publishing results, nor should it have become the sole arbiter of supporting or refuting a hypothesis derived from a scientific theory [40,41,42]. Most psychological research entails gathering empirical evidence to support inferences to an explanatory theory or model. Rarely are psychologists truly interested in drawing inferences to population parameters. One need only search published studies for random—or even representative—samples to find support for this statement. Without extreme attention to sampling methods, inferences to population parameters are hazardous and ill-advised. Strictly speaking, without random sampling, *p*-values from NHST—which serve as the gatekeepers to publication—are in fact uninterpretable [43].

While not a strict litmus test, then, psychologists can ask a simple question, “Is drawing a random sample critically important for my proposed research?” If the answer is in the affirmative, then it is likely their intention is to seek inferences to population parameters. As the vast majority of psychological research is conducted on undergraduate university students who were not randomly selected from their own universities nor their wider communities [44], however, it is safe to conclude that the answer to the question will routinely be negative. Consequently, the inference sought by most research psychologists will be the inference to best explanation, and the studies above show how they can reason about and analyze their data in a manner congruent with this inference.

Antidotes to the other bad habits require psychologists to devise novel ways to model psychological phenomena and to analyze their data in ways which are optimally aligned with these models. The second study above regarding enhanced memory demonstrated how such novel models can be constructed and tested. Drawing ellipses to represent latent variables and boxes to represent observed variables and then connecting those boxes together with arrows is *prima facie* wholly inadequate for modeling the complexities of human behavior, a point also made by Tryon [45]. Such variable-based models do not offer the tools necessary to make important distinctions between the various structures and processes comprising the human activity under investigation. By contrast, even the simple integrated models presented above provided the means for differentiating between various cognitive processes and distinct types of causes. Additional examples of such models can be found in the work of Grice and his colleagues [11,46,47,48], Powers [49,50], and Cevasco and Marmolejo-Ramos [51].

Importantly, these models describe the causal structure of human action at the level of the person. As shown in all of the studies above, the level of analysis most appropriate for psychological research is often the individual rather than the aggregate [52,53]. Experimental statistical analyses have long dominated psychological research, but most of these methods are tailored to the analysis of means, variances, and covariances in the context of the General Linear Model and NHST. With integrated models, the analysis plan is one that searches for theory-relevant patterns among the observations themselves. Once posited, tested, and replicated, these patterns can be scrutinized and developed systematically over time. The contrast between the traditional statistical and integrated modeling approaches can be seen in a recent target article and perceptive commentary within the Neuroscience literature ([54]; with commentary by Ashton [55]). As psychologists rarely work with established units of measure for continuously structured qualities, the pattern searching and testing approach is moreover likely to entail working with countable and ordered observations and logical relations. Today, psychologists can choose optimal nonparametric and/or algorithmic/computational statistical methods of analyses rather than conventional statistical model-based parametric methods for the lion’s share of their analyses. Breiman [13] made this point very clearly 16 years ago, yet the majority of psychologists persist with the old ways of analysis.

## 3. Conclusions

In conclusion, we return to St. Thomas Aquinas who considered a *habitus* to be a quality which insures that a person will perform an action with ease, accuracy, and consistency. St. Thomas moreover drew a distinction between *entitative* and *operative* habits. The former are regarded as naturally acquired, such as an individual with an impulsive temperament or an individual who is inclined toward superior athletic performance. Such habits are often recognized at a young age and are not easily modifiable. Operative habits, by comparison, are acquired through education, experience, and repetition (i.e., practice) and are therefore relatively open to change. St. Thomas considered science to be a *habitus* of the operative species and therefore modifiable. With this in mind we are hopeful that, despite the many cultural forces opposing significant changes in their research habits, psychologists will find the courage to challenge existing methodologies and analysis techniques. Change need not be viewed as a threat, but rather as a fact of the dramatically evolving face of modern science, “As argued by Thagard [56], science has changed out of recognition over the course of the twentieth century. Whereas, the early days of the century witnessed the establishment of the now traditional disciplines and divisions, some of which have been retained in the current curricula of universities, many of the critical advances in science and technology reflected migration to the boundaries of the established disciplines, as, like memes, they embarked on new inter-disciplinary journeys of their own. These transformations are particularly clear in the new and rapidly changing sciences, and the industries behind them, underwater target detection and forensic science being two obvious examples. It is also very clear in medical science and in medical training, where the nature and application of knowledge are undergoing similar transformations” [57] (p. 17).
